# Supplementary figures and images for: CGRP overexpression does not alter depression-like behavior in mice
Source: PeerJ. 2021 Jul 2;9:e11720. doi: 10.7717/peerj.11720 (PMC8256807; doi:10.7717/peerj.11720)

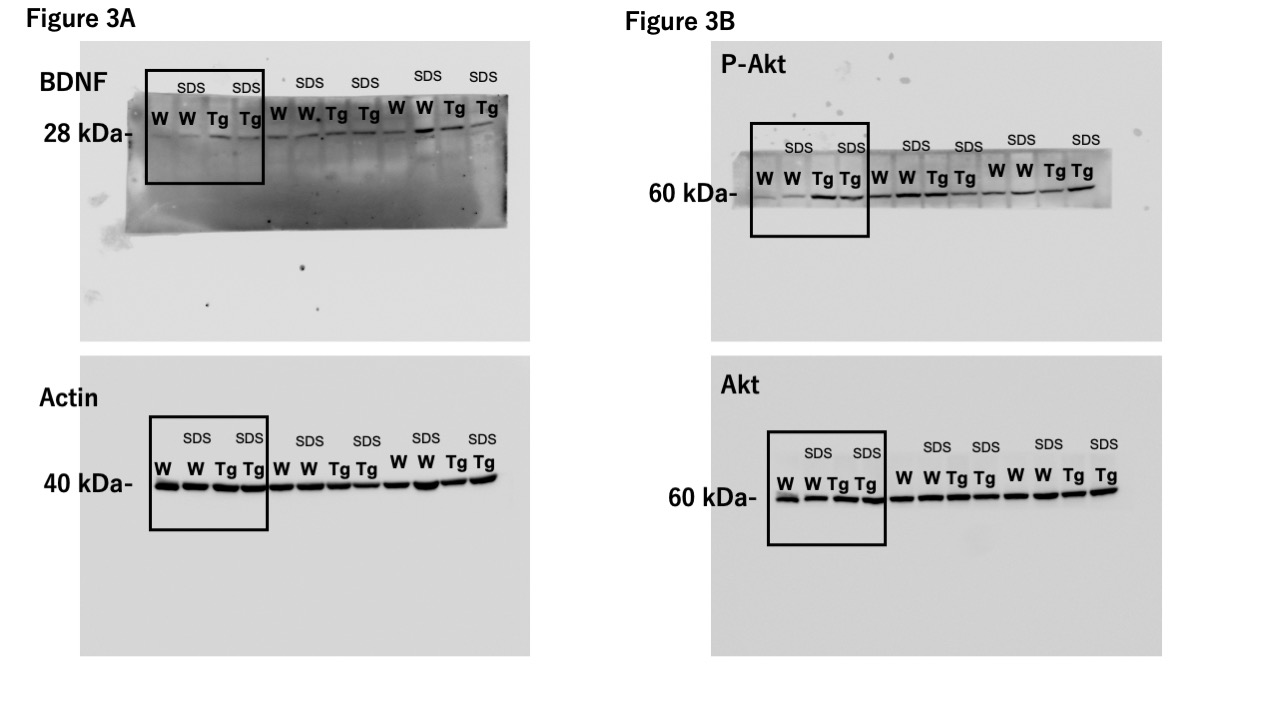

Supplement: Supplemental Information 5 — Upper gel is BDNF. Bottom gel is Actin. [file peerj-09-11720-s005.jpeg]

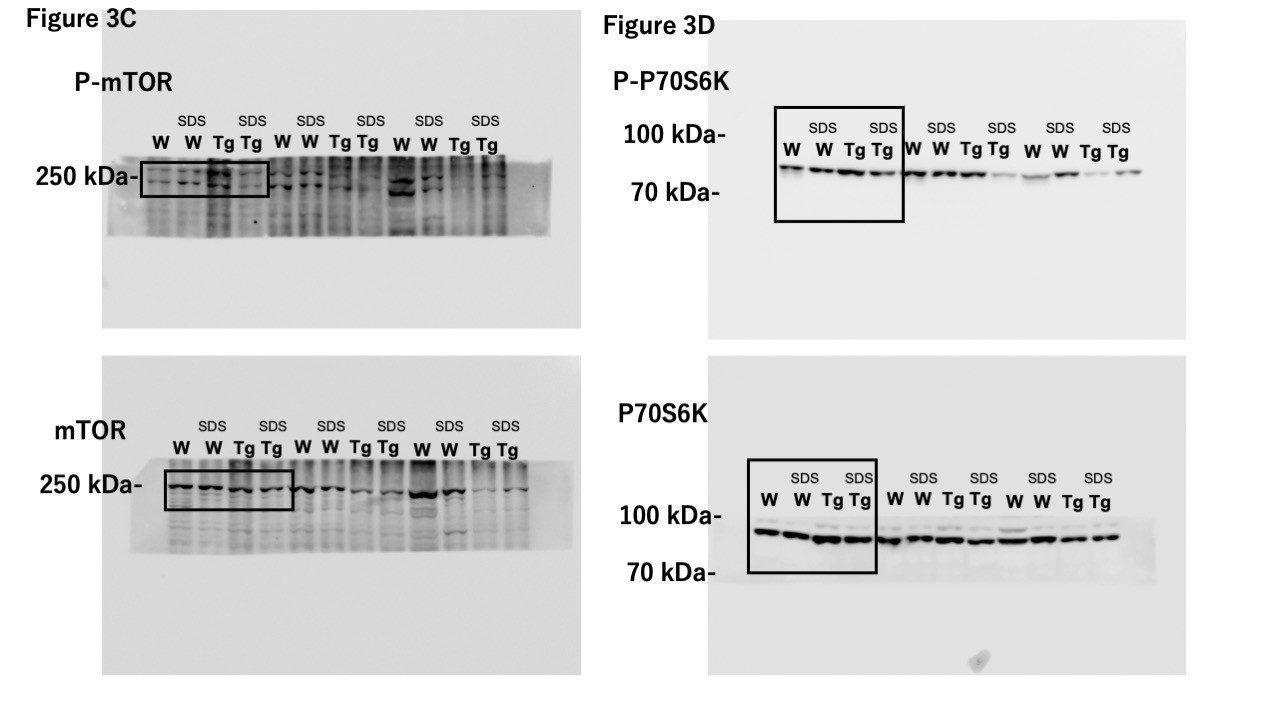

Supplement: Supplemental Information 6 — Upper gel is phosphorylated-Akt. Bottom gel is Akt. [file peerj-09-11720-s006.jpeg]
